# A computational method for estimating the PCR duplication rate in DNA and RNA-seq experiments

**DOI:** 10.1186/s12859-017-1471-9

**Published:** 2017-03-14

**Authors:** Vikas Bansal

**Affiliations:** Department of Pediatrics, School of Medicine, University of California San Diego, 9500 Gilman Drive, 92093 La JollaCA, USA

**Keywords:** PCR duplicates, High-throughput sequencing, Mathematical modeling, Heterozygosity, RNA-seq, Natural duplicates

## Abstract

**Background:**

PCR amplification is an important step in the preparation of DNA sequencing libraries prior to high-throughput sequencing. PCR amplification introduces redundant reads in the sequence data and estimating the PCR duplication rate is important to assess the frequency of such reads. Existing computational methods do not distinguish PCR duplicates from “natural” read duplicates that represent independent DNA fragments and therefore, over-estimate the PCR duplication rate for DNA-seq and RNA-seq experiments.

**Results:**

In this paper, we present a computational method to estimate the average PCR duplication rate of high-throughput sequence datasets that accounts for natural read duplicates by leveraging heterozygous variants in an individual genome. Analysis of simulated data and exome sequence data from the 1000 Genomes project demonstrated that our method can accurately estimate the PCR duplication rate on paired-end as well as single-end read datasets which contain a high proportion of natural read duplicates. Further, analysis of exome datasets prepared using the Nextera library preparation method indicated that 45–50% of read duplicates correspond to natural read duplicates likely due to fragmentation bias. Finally, analysis of RNA-seq datasets from individuals in the 1000 Genomes project demonstrated that 70–95% of read duplicates observed in such datasets correspond to natural duplicates sampled from genes with high expression and identified outlier samples with a 2-fold greater PCR duplication rate than other samples.

**Conclusions:**

The method described here is a useful tool for estimating the PCR duplication rate of high-throughput sequence datasets and for assessing the fraction of read duplicates that correspond to natural read duplicates. An implementation of the method is available at https://github.com/vibansal/PCRduplicates.

**Electronic supplementary material:**

The online version of this article (doi:10.1186/s12859-017-1471-9) contains supplementary material, which is available to authorized users.

## Background

High-throughput sequencing (HTS) technologies have found widespread use in genomics, transcriptomics and epigenomics. PCR amplification is an important step in virtually all library preparation protocols for high-throughput sequencing technologies [1, 2]. In the standard Illumina library preparation protocol, after universal adapters are ligated to the pool of DNA fragments, PCR amplification is done in order to enrich for fragments that have adapters ligated on both ends and can be sequenced successfully [3, 4]. Hybridization-based target enrichment protocols used for whole exome sequencing as well as experiments that start from low quantities of input material also require PCR amplification.

If the number of unique DNA template molecules in the initial library is small or if there are steps in the library preparation that reduce the number of distinct DNA fragments, some fragments can end up being sequenced multiple times. These so called “PCR duplicates” correspond to redundant information, i.e. copies of the same DNA fragment. A high frequency of PCR duplicates is undesirable since it reduces the effective sequencing coverage of the experiment. A high PCR duplication rate cannot be overcome simply by sequencing to higher coverage. Rather, it indicates the need to modify the library preparation to improve the complexity of the sequencing library. For large-scale sequencing projects involving multiple samples, it is important to identify outlier samples with a high PCR duplication rate that can bias the joint analysis of the sequence data. Therefore, for many reasons, it is of great interest to estimate the PCR duplication rate of high-throughput sequence datasets.

Read duplicates can be identified after sequencing using alignment of reads to a reference genome. Groups of reads that map to the same genomic coordinates (both forward and reverse reads for a paired-end sequencing protocol) on the reference sequence and are also identical in sequence (allowing for a few sequencing errors) represent clusters of read duplicates [5]. Read duplicates can correspond to technical duplicates such as those due to PCR amplification (and optical duplicates [6]) or natural read duplicates. ‘Natural’ read duplicates (also referred to as sampling duplicates) arise due to the saturation of the space of possible start and end positions for DNA fragments. For whole genome and exome sequencing experiments using paired-end reads, almost all read duplicates correspond to technical duplicates. However, in many applications, natural read duplicates can represent a significant fraction of the read duplicates [7]. For example, in RNA-seq experiments, some regions of the genome (highly expressed genes) have much higher coverage than others and as a result a large fraction of the duplicate reads represent independent fragments sampled from such regions. Sequencing experiments that utilize single end sequencing also show a high frequency of natural read duplicates since reads with identical 5’ mapping coordinates cannot be distinguished from PCR duplicates [8]. In such scenarios, it is important to determine the fraction of read duplicates that are due to PCR amplification since removing all read duplicates can bias downstream analysis such as estimation of gene expression values.

Natural read duplicates can be distinguished from PCR duplicates using molecular methods [9–13]. These methods add a unique molecular identifier (UMI) or a random barcode to each DNA fragment prior to PCR amplification and sequencing. Post sequencing, natural read duplicates are unlikely to share the UMI while PCR duplicates will have identical alignment coordinates and UMI. Although, these methods have been shown to improve the accuracy of variant calling in DNA-seq experiments [9] and expression quantification in mRNA-seq experiments [10], they require specialized modifications to the library preparation protocols and are not routinely used. Recognizing the high frequency of natural read duplicates in some sequencing experiments, computational methods to model the probability of natural read duplicates in DNA and RNA sequencing experiments have also been developed [7, 14]. However, these methods do not provide an explicit estimate of the PCR duplication rate.

In this paper, we describe a novel computational method to estimate the PCR duplication rate of a high-throughput sequence dataset that accounts for natural read duplicates. Our method utilizes reads that overlap heterozygous variants sites to estimate the relative proportion of PCR duplicates and natural read duplicates. We present a mathematical model for modeling read duplicates that is used to estimate the relative proportion of PCR and natureal read duplicates in sequence data. Using simulated data as well as exome datasets from the 1000 Genomes Project [15], we demonstrate the accuracy of our method in estimating the PCR duplication rate from datasets even with a high frequency of natural duplicates. Further, we analyze RNA-seq data on samples from the 1000 Genomes project [16] to demonstrate that only a small fraction (5–30%) of read duplicates observed in RNA-seq data are due to PCR amplification.

## Results and discussion

### Overview of method

**Fig. 1:**
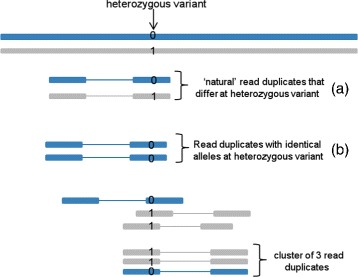
Illustration of paired-end reads covering a heterozygous SNV (reference allele is denoted by 0 and the variant allele as 1) in a diploid genome. The reads can be grouped into clusters of different sizes based on their alignment coordinates. Two reads that start and end at the same position but carry different alleles (0 and 1) at the heterozygous site (**a**) are highly likely to correspond to natural duplicates, i.e. independent DNA fragments. In contrast, a pair of read duplicates that have identical alleles at the heterozygous site (**b**) could correspond to PCR duplicates or natural duplicates

The first step in the analysis is to identify all groups or clusters of read duplicates such that all reads in each cluster have identical outer mapping coordinates. Each cluster of read duplicates is a combination of natural read duplicates (independent DNA fragments) and PCR duplicates. A cluster of two read duplicates can correspond to either (i) one independent DNA fragment (and a PCR duplicate) or (ii) two independent DNA fragments. We observe that PCR duplicates represent copies of a single DNA molecule and are expected to have identical alleles at a heterozygous variant site (unless an error occurs during sequencing or PCR amplification). In contrast, a pair of natural read duplicates will have the same allele if they are sampled from the same chromosome and show opposite alleles at a heterozygous site if they are sampled from the opposite homologous chromosomes (see Fig. 1 for an illustration). Therefore, analysis of the counts of clusters with matching or opposite alleles at heterozygous sites can be used to estimate the proportion of natural duplicates among duplicate clusters of size 2.

Assuming equal likelihood of sampling a read from one of the two chromosomes, half of the natural read duplicates are expected to have opposite alleles at a heterozygous site. Therefore, if *C*_2_ is the total number of clusters of size 2 that overlap heterozygous variant sites and *C*_21_ be the subset of clusters with opposite alleles, the expected number of clusters of size 2 that correspond to natural read duplicates and PCR duplicates are 2·*C*_21_ and *C*_2_−2·*C*_21_ respectively. These estimates can be used to estimate the average number of unique DNA fragments for clusters of size 2 as:

1 $$ U_{2} = \frac{1 \cdot (C_{2} - 2C_{21}) + 2 \cdot 2 C_{21}}{C_{2}}   $$

**Fig. 2:**
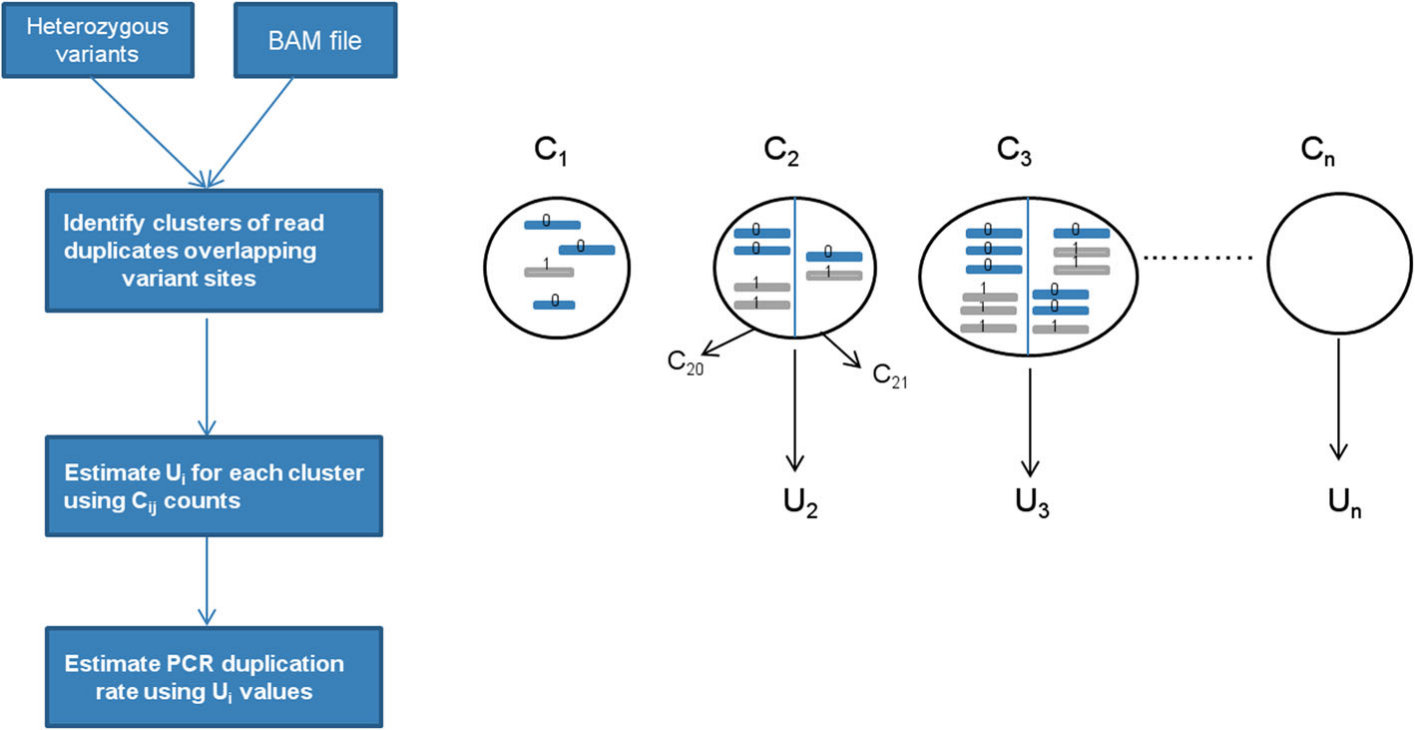
Overview of computational method for estimating the PCR duplication rate using clusters of duplicate reads that overlap heterozygous variant sites. *C*_*i*_ corresponds to the clusters of read duplicates with *i* reads and *U*_*i*_ is the average number of unique DNA fragments for clusters of size *i*

While *U*_2_ gives a good indication of the relative frequency of PCR duplicates and natural duplicates in a sequence dataset, in order to estimate of the PCR duplication rate, we need to calculate *U*_*i*_ for larger cluster sizes (see Fig. 2 for an overview of the method). To analyse clusters of size greater than two, we utilize a mathematical model that uses basic probability and counting arguments to estimate the fraction of duplicate clusters with different number of unique DNA fragments (see Methods for details).

### Accuracy of the method on simulated data

To assess the accuracy of the method for estimating PCR duplication rate, we used simulated data that was generated using paired-end exome data from a single sample (HG00110) sequenced in the 1000 Genomes Project. Our goal was to assess the accuracy of our method for estimating the PCR duplication rate in the presence of natural read duplicates. Therefore, we simulated datasets with both PCR duplicates and natural read duplicates (see Methods for details of simulation procedure).

**Fig. 3:**
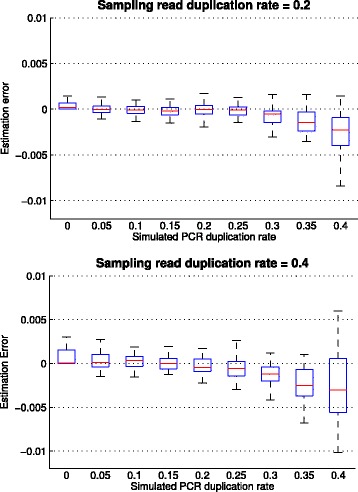
Box-plot showing the error in the estimation of the PCR duplication rate using our method on simulated data with varying levels of PCR duplicates (0 to 0.4). Data was simulated with a fixed sampling read duplication rate (plots shown for values of 0.2 and 0.4). For each combination of values, 50 simulated datasets were used to assess the error of the estimated PCR duplication rate

The estimated PCR duplication rate using our method was highly accurate (*r* ^2^=0.9996 between the simulated and estimated PCR duplication rate). The error in the estimation of the PCR duplication rate increased as the PCR duplication rate increased from 0 to 0.4 (Fig. 3) and was greater for higher values of the sampling duplication rate (0.4 vs 0.2, Fig. 3). We also observed that our method tended to slightly underestimate the PCR duplication rate as the PCR duplication rate increased (−0.64*%* for PCR duplication rate = 0.4 and sampling duplication rate = 0.4, Fig. 3). Overall, our method was able to estimate the PCR duplication rate even in the presence of a high frequency of natural read duplicates with a low mean absolute percentage error (less than 1.1% across all simulations).

PCR amplification is non-uniform and DNA fragments with a high or low GC content are less likely to be amplified [17]. To assess the impact of non-uniform PCR duplication rate on the accuracy of our method, we simulated data with a PCR amplification rate that varied as a function of the GC content of each DNA fragment (estimates were obtained from empirical sequence data [17]). We simulated 50 datasets with a natural read duplicate rate of 0.2 and a randomly selected PCR duplication rate (range 0 to 0.5). Comparison of the simulated and estimated PCR duplication rates showed that our method was able to accurately estimate the PCR duplication rate (correlation coefficient = 0.999 and mean absolute difference = 0.0023).

### Accuracy of the method on real exome data

To assess the ability of our method to estimate the PCR duplication rate on DNA sequence datasets, we utilized a sample set of 40 Illumina exome datasets from the 1000 Genomes Project [15]. For each individual, a set of heterozygous SNVs identified using the GATK UnifiedGenotyper [5] tool was used for estimating the PCR duplication rate. For each individual dataset, the PCR duplication rate was estimated in paired-end mode and single-end mode, ie. by ignoring the insert length information. Single end sequence data shows a much higher frequency of natural duplicate reads since reads that start at the same genomic position after alignment cannot be distinguished further in the absence of fragment length information. Indeed, the read duplication rate of the SE reads for each sample (using read1 of each paired-end read) was on average 5.8 times greater than the read duplication rate for the PE reads (Additional file 1: Table S1). However, PCR duplication rate of the dataset should be independent of whether we utilize PE reads or only SE read information.

**Fig. 4:**
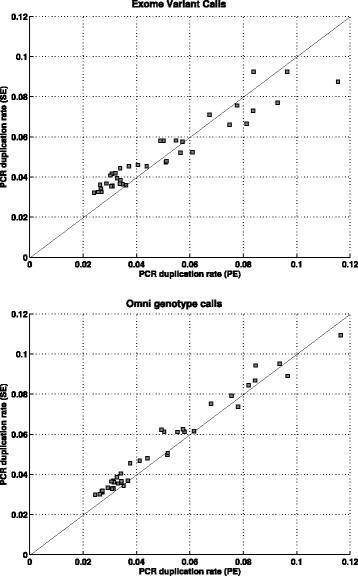
Comparison of the estimated PCR duplication rate on 40 exome datasets from the 1000 Genomes Project analyzed as paired-end (PE) reads and single-end (SE) reads. The two plots correspond to the analysis using exome variant calls and Omni genotype calls. For visual clarity, two outlier samples with a high PCR duplication rate (>0.12) are not shown

We found that the estimated PCR duplication rate from the data treated as SE reads (SE-PCR duplication rate) was highly concordant (*r* ^2^=0.977, *p*-value = 5.3×10^−27^ and mean absolute difference = 0.0073) with the PCR duplication rate estimated from the data analyzed as PE reads (Fig. 4, top panel). In contrast, the correlation between the SE read duplication rate and the PE read duplication rate was much lower (*r* ^2^=0.542). Further, both the SE and PE PCR duplication rate estimates were slightly lower (1–9%) than the PE read duplication rate which is an upper bound for the PCR duplication rate. These results demonstrated that our method can accurately estimate the PCR duplication rate on real sequence datasets even in the presence of a high proportion of natural read duplicates.

To compare the performance of our method with existing computational methods for analyzing the complexity of sequencing libraries, we analyzed the PE and SE read data for one exome dataset (HG00110) using the PreSeq method [18]. This method was developed to estimate the complexity of sequencing libraries with the goal of predicting the benefit of increasing the sequencing depth for a library. The fraction of duplicate reads estimated using this method for the PE (0.057) and SE (0.321) datasets was identical to that based on the analysis of read duplicates and not informative about the PCR duplication rate. This was not surprising since this method needs UMIs to distinguish between PCR duplicates and natural read duplicates.

We also analyzed an Illumina exome dataset for a HapMap individual(NA12812) from Bainbridge and colleagues [19]. The authors had compared the read duplication rate for this exome capture dataset using PE and SE reads. They found that the read duplication rate for the PE dataset was 7.61%. In comparison, the read duplication rate for single reads was observed to be almost four times greater (27.6%). Using our method, the estimated PCR duplication rate on the SE reads (read1) was 7.23%, very similar to the PCR duplication rate estimated from the PE reads (7.29%).

### Robustness of the PCR duplication rate estimate to variant calls

Our method only requires a subset of the heterozygous variants in an individual genome to estimate the PCR duplication rate. In the previous section, the PCR duplication rate for the exome datasets was estimated using heterozygous variants identified from the exome data itself. To assess the robustness of the estimate of the PCR duplication rate to the choice of the variants used, we estimated the PCR duplication rate using Illumina Omni array genotype calls (see Methods for details) for these samples. For each sample, we estimated the PCR duplication rate on the exome reads treated as PE and SE reads respectively using the set of heterozygous genotypes from the Omni genotype data. The mean number of heterozygous variant sites (covered by at least 8 reads) from the Omni genotype data was 18,640 across the 40 samples.

The estimates of the PE-PCR duplication rate using the Omni genotypes were tightly correlated with the estimates obtained from the exome variant calls (*r* ^2^=1.0 and mean absolute difference =0.0005). The estimates of the SE-PCR duplication rate using the exome variant calls and the Omni genotypes were also strongly correlated (*r* ^2^=0.982). These results indicate that the estimate of the PCR duplication rate is consistent between the two sets of heterozygous variants. Further, the concordance between the estimates of the PE-PCR duplication rate and the SE-PCR duplication rate on the 40 exome datasets (*r* ^2^=0.987 and mean absolute difference =0.005, Fig. 4, bottom panel) was marginally better than the concordance between the estimates using exome variant calls (*r* ^2^=0.977 and mean absolute difference =0.0074, Fig. 4, top panel).

Next, to assess the robustness of the PCR duplication rate estimate to the size of the variant set, we analyzed exome data for one sample (HG00110, SE reads) using subsets of the heterozygous variant calls generated from exome data. The full set of heterozygous variants included 14,741 SNVs and the estimated PCR duplication rate was 0.0582. The mean of the estimated PCR duplication rate using 50% of the variants was 0.0581±0.0009 (standard error estimated using 50 random subsets). The mean estimate decreased slightly to 0.0576±0.0018 when using 20% of the variants. Although, the PCR duplication rate was underestimated for smaller sets of variants due to lack of sufficient clusters counts for large clusters (*k*>8 for 20% of variants), the decrease in the estimate was small (less than 1% of the estimated value on the full set of variants). This demonstrated that our method can estimate the PCR duplicate rate on DNA sequence data using a small number of variants.

### Frequency of natural read duplicates across exome capture protocols

A number of different methods have been developed for performing human whole-exome capture experiments [20]. These include the Agilent SureSelect, NimbleGen SeqCap, Illumina TruSeq and Illumina Nextera. Among these, the Nextera library preparation method fragments DNA and adds the adapters to the DNA fragments in a single step using a transposase [21, 22]. All other exome capture protocols fragment DNA by sonication. The 40 exome datasets in the 1000 Genomes project were obtained using the Agilent SureSelect and the NimbleGen SeqCap capture methods. The PCR duplication rate of these datasets was 1–9% lower than the read duplication rate across the 40 exome datasets (Additional file 1: Table S1). This indicated that only a small fraction of the read duplicates correspond to natural read duplicates. The low frequency of natural read duplicates was not surprising since in paired-end (PE) sequencing libraries prepared with random DNA fragmentation, the probability that two independent DNA fragments have identical starting position and fragment length is low.

The transposase-mediated fragmentation in the the Nextera library preparation method can introduce sequence dependent biases in the data compared to standard methods of fragmentation [21]. Although the impact of non-uniform fragmentation on the uniformity of sequence coverage is negligible, it has the potential to increase the frequency of natural read duplicates. To assess this, we analyzed 12 Nextera exome capture datasets (4 replicates each for three individuals NA12878, NA12891 and NA12892) from the the Illumina BaseSpace repository. The read duplication rate for the 12 datasets was low (4–6%) (Additional file 1: Figure S2). However, the estimates of the PCR duplication rate using our method were even lower (1.5–2%) and indicated that 55–70% of the read duplicates correspond to natural read duplicates. This was significantly higher than than the proportion of natural read duplicates (<10%) in exome datasets from the 1000 Genomes project and likely reflects fragmentation bias in the Nextera library preparation method. Although the Nextera library preparation approach has several advantages compared to standard methods including speed and low input requirements, our results demonstrate that it results in a high proportion of natural read duplicates compared to other exome enrichment protocols.

### Analysis of PCR duplication rates for RNA-seq data

Sequencing of complementary DNA (cDNA) by high-throughput sequencing technologies provides quantitative information about the abundance (and sequence) of mRNA transcripts and is becoming the method of choice for analyzing gene expression and RNA splicing [23]. Compared to DNA sequence datasets, a high rate of read duplicates are typically observed in RNA-seq datasets. However, unlike DNA sequence datasets, a significant fraction of these read duplicates likely represent independent fragments that originate from transcripts with high expression levels rather than PCR duplicates. The frequency of such natural duplicates is further increased due to fragmentation bias in RNA-seq library preparation resulting in a non-uniform distribution of fragments across each transcript [24, 25]. Unlike for DNA sequencing studies where read duplicates are removed prior to variant calling, there is no clear consensus on how to deal with duplicate reads in RNA-seq data. On one hand, removal of all read duplicates prior to expression quantification can result in underestimation of expression levels for highly expressed genes. On the other hand, not accounting for PCR duplicates can inflate read counts and potentially affect the accuracy of differential gene expression analysis.

**Fig. 5:**
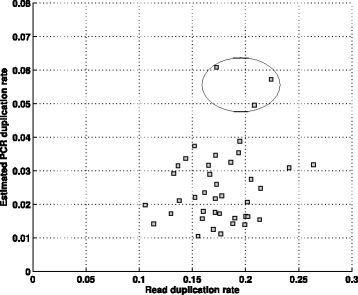
Comparison of the read duplication rate and the estimated PCR duplication rate for 40 RNA-seq samples from the Geuvadis project. Three samples with much higher PCR duplication rates than the remaining samples are highlighted

While it is generally accepted that a significant proportion of read duplicates in RNA-seq datasets are not due to PCR amplification, there is little quantitative analysis of the PCR duplication rate in RNA-seq datasets. To assess the ability of our method to estimate the PCR duplication rate on RNA-seq data, we utilized RNA-seq data from lymphoblastoid cell lines of individuals from the 1000 Genomes Project that was generated by the Geuvadis project [16]. We estimated the PCR duplicate rate on RNA-seq data for the same set of 40 samples for which we analyzed exome data previously. Variant calls for estimating the PCR duplication rate were derived from the exome data. We found that the PCR duplication rate for these RNA-seq datasets was quite low (1–6%) in comparison to the observed read duplication rate which varied between 10 and 26% (Fig. 5). This indicated that the vast majority (70–95%) of read duplicates in the RNA-seq datasets are not due to PCR amplification. Next, to assess the robustness of the PCR duplication rate estimates to variant calls, we utilized heterozygous variant sets for each individual obtained from the Omni genotype data. We found that the two sets of PCR duplication rate estimates (exome calls vs Omni genotype calls) were highly concordant with correlation coefficient *r* ^2^=0.96 and mean absolute difference =0.0027 (see Additional file 1: Figure S3).

For RNA-seq experiments, the frequency of natural read duplicates is expected to increase with increasing sequence coverage. On the other hand, the PCR duplication rate depends on the complexity of the sequencing library and should be more or less independent of the number of reads. Indeed, while the read duplication rate due to sampling (read duplication rate - estimated PCR duplication rate) was strongly correlated with the total number of reads per sample (*r* ^2^=0.495, *p*-value = 0.001), there was no correlation between the estimated PCR duplication rate and number of reads per sample (*r* ^2^=−0.177, *p*-value =0.274). In the Geuvadis project [26], the authors analyzed 465 RNA-seq datasets and used a number of metrics including the read duplication rate to identify outlier samples. Of the 40 samples analyzed, the PCR duplication rate for three samples (mean =0.053, see Fig. 5) was more than 2-fold higher than the average PCR duplication rate for the remaining samples (0.023). It is noteworthy that the read duplication rate for these outlier samples was not higher than the remaining samples. Such ‘outlier’ samples with a high PCR duplication rate should be excluded from the joint analysis of multiple RNA-seq datasets to avoid biasing downstream results. In addition, some pairs of samples had identical read duplication rates (∼18%) but very different PCR duplication rates (Fig. 5). These results illustrate the utility of the PCR duplication rate estimate provided by our method as an independent metric to assess the quality of RNA-seq datasets.

## Conclusions

PCR amplification is a necessary step in the preparation of DNA sequencing libraries for most high-throughput sequencing instruments. However, PCR amplification is inherently biased and introduces artifacts into the sequence data including duplicate reads. Estimating the PCR duplication rate after sequencing can provide important information about the quality of the sequence dataset and the complexity of the library. While estimating the read duplication rate from aligned sequence reads is straightforward, accurate estimation of the PCR duplication rate requires estimating the fraction of read duplicates that represent natural read duplicates. Previous computational methods have attempted to model the probability of natural read duplicates [7, 14] in DNA and RNA sequencing experiments. In this paper, we have presented a novel approach for estimating the PCR duplication rate that utilizes heterozygous variants in a diploid genome to estimate the fraction of natural read duplicates. To the best of our knowledge, the method presented in this paper is the first computational method that aims to estimate the average PCR duplication rate of high-throughput DNA sequence datasets while accounting for natural read duplicates. We have demonstrated its accuracy using both simulated data and exome data from the 1000 Genomes Project. We have shown that it can even estimate the PCR duplication rate with high accuracy from datasets with a high frequency of natural read duplicates.

We also analyzed RNA-seq data from a population study of human transcriptomes to demonstrate that the vast majority of read duplicates (70–95%) in RNA-seq data are not due to PCR amplification. Our results are consistent with the observation of [16] that “majority of duplicate reads in a high-quality mRNA experiment are due to saturation of the read mapping space driven by real biology of high expression levels”. If the PCR duplication rate is low, not removing read duplicates prior to estimating transcript expression levels is unlikely to bias the results. However, not all RNA-seq datasets may have a low level of PCR duplicates. Therefore, a computational estimate of the PCR duplication rate provided by our method can be utilized to determine whether PCR duplicates should be ignored or not. It can also be used to identify outlier samples with a high PCR duplication rate relative to other samples in large-scale human transcriptome studies. We note that the estimation of the PCR duplication rate from RNA-seq data is more challenging compared to that from DNA-seq data due to the high frequency of read duplicates, strong allele bias due to alignment of spliced reads and the need for an independent set of heterozygous variants.

The mathematical model underlying our method makes very few assumptions about the PCR amplification process or how natural duplicates are generated. It is agnostic to the distribution of insert lengths and start sites for DNA fragments in the sequencing library. However, it is designed to estimate the average PCR duplication rate and cannot be applied to datasets derived from haploid genomes or datasets with a small number of heterozygous variants. The model can potentially be extended to incorporate sequencing error rates and allele bias to further improve the accuracy of the estimates. The computational method that utilizes this model to estimate the PCR duplication rate can be applied to analyze both DNA and RNA-seq datasets. It is computationally efficient (an exome dataset with 50 million PE reads can be processed in less than 5 min on a standard workstation) and accepts input files in standard BAM and VCF formats. It requires no additional sequencing or modifications to the library preparation protocol and gives useful information about the PCR duplication rate that is not provided by existing methods.

## Methods

### Detection of clusters of duplicate reads

Using the sorted list of aligned reads, all groups or clusters of read duplicates were identified such that all reads in each cluster have identical outer mapping coordinates (5’ and 3’ for paired-end reads). To account for the incomplete alignment of some reads by some short read alignment tools (soft-clipping), the alignment coordinates (start and end) of partially aligned reads were adjusted. For single-read analysis, a pair of single-end reads were identified as duplicates if they had identical 5’ position and were aligned to the same strand. From the read clusters, the distribution of cluster counts: $({\hat C}_{1}, {\hat C}_{2}, \ldots)$ was obtained where ${\hat C}_{i}$ is the number of clusters with *i* read duplicates. For the subset of clusters that overlap heterozygous variant sites, the allele for each read in a cluster was determined using the read sequence. Base calls with low quality values (default threshold of 20) were were not utilized for estimation.

### PCR duplication rate

The read duplication rate of a sequence dataset with a total of *R* reads is defined as: $ 1- {\sum _{i} {\hat C}_{i}}/{R}$. If *U*_*i*_ denotes the average number of unique DNA fragments per cluster of size *i*, the PCR duplication rate can be estimated as:

2 $$ 1.0 - \frac{\sum_{i=1}^{n} U_{i} {\hat C}_{i}}{R}   $$

### Estimating the PCR duplication rate using heterozygous sites

In order to estimate the PCR duplication rate, we need to estimate the expected value of *U*_*i*_ for 1<*i*≤*n*. A cluster of read duplicates of cardinality *i* can have *j* (1≤*j*≤*i*) independent DNA fragments. A subset of the duplicate read clusters overlap heterozygous variant sites (Fig. 1). Such clusters can be further categorized based on the alleles observed at the heterozygous sites in the reads for each clusters. At each heterozygous site, we denote the two alleles as 0 and 1 (bi-allelic site in diploid individual). We denote by *C*_*i*_ the number of clusters of size *i* that overlap a heterozygous site and by *C*_*ik*_, the number of such clusters for which *k* reads in the cluster match one allele and *i*−*k* reads match the second allele. Note that the counts *C*_*ik*_ represent the data that we obtain from the aligned sequence reads. Further, we denote by *C*_*i*_(*j*) the (unknown) number of clusters of size *i* with *j* independent DNA fragments. Therefore, we can write

$$C_{i} = \sum\limits_{k=0}^{i/2} C_{ik} \text{ and } C_{ik} = \sum\limits_{j=1}^{i} C_{ik}(j) $$

Our method utilizes the observed counts *C*_*ik*_ to estimate the relative proportions of natural duplicates and PCR duplicates for each cluster size. The two key equations for all clusters of size *i* (*i*≥2) are as follows:

3 $$ C_{i0} = C_{i0}(1) + \ldots + C_{i0}(i)   $$

4 $$ C_{i} = C_{i}(1) + \ldots + C_{i}(i)   $$

For a cluster of size *i* with *j* independent DNA fragments, there are 2^*j*−1^ potential configurations of the two alleles (we consider the symmetric configurations 0^*a*^1^*b*^ and 0^*b*^1^*a*^ as identical). Since, we assume that the probability of sampling the two alleles at a heterozygous site is equal, each of these configurations are equally likely and we can approximate *C*_*i*_(*j*) as 2^*j*−1^ *C*_*i*0_(*j*). Using this approximation in Eq. (4), we get

5 $$ C_{i} \approx C_{i0}(1) + \ldots + 2^{i-1} C_{i0}(i)   $$

Estimating *U*_2_ and *C*_21_ for clusters of size 2 is straightward using the two Eqs. (3 and 5). For clusters of size 3, we have three unobserved variables (*C*_30_(1), *C*_30_(2) and *C*_30_(3)) and only two expressions ((3) and (5)). We use elementary probability to relate *C*_30_(2) with the counts *C*_20_(2) and *C*_20_(1) estimated from clusters of size 2. Note that a cluster of size 2 with one independent DNA fragment is the result of a single PCR duplication event. Similarly, a read cluster of size 3 with 2 independent DNA fragments results from a single PCR duplication of one of the two DNA fragments. Let *p* be the probability that an independent DNA fragment undergoes a single PCR duplication event. This probability does not depend on whether the DNA fragment has a natural read duplicate or not. Note that the probability of PCR amplification is not uniform across DNA fragments and depends on the GC content and length of the fragments. Here, *p* corresponds to the average rate of PCR duplication across all reads. Therefore, we can write:

$${}\frac{C_{20}(1)}{C_{1}} \approx \frac{p}{1-p} \text{ and } \frac{C_{30}(2)}{C_{20}(2)} \approx \frac{2p(1-p)}{(1-p)(1-p)} = 2 \frac{p}{1-p} $$

It follows that

$$ C_{30}(2) \approx 2 \left[\frac{C_{20}(1)}{C_{1}}\right] C_{20}(2) $$

From the analysis of clusters of size 2, we already have estimates for *C*_20_(1) and and *C*_20_(2). Using the estimated value for *C*_30_(2) in Eq. (3) (for *i*=3) and (5), we can calculate estimates for *C*_30_(1) and *C*_30_(3). From these estimates, it is straightforward to estimate *U*_3_. The analysis for clusters of size 3 can be generalized to clusters of arbitrary size. For this, we define

6 $$ {\lambda}_{i} = \frac{C_{i0}(1)}{C_{1}}  $$

where *C*_*i*_(1) corresponds to read clusters of size *i* that represent a single independent DNA fragment and *i*−1 PCR duplicates. By definition, *λ*_1_=1. Consider a cluster of size *i* with matching alleles at all reads in the cluster. We note that there is a one-to-one correspondence between the different possible ways in which a cluster of size *i* can result from the PCR amplification of *j* unique DNA fragments (1≤*j*≤*i*) and the integer partitions of *i*. For example, the integer partitions of 4 are {[4],[3,1],[2,2],[2,1,1],[1,1,1,1]}. The partition [4] corresponds to a cluster with a single DNA fragment and three PCR duplicates. Similarly, the partition [1,1,1,1] corresponds to a cluster with four independent DNA fragments. The expected frequency of clusters corresponding to each partition can be estimated using *λ*_*i*_’s and the counts for smaller size clusters. In general, the expected frequency of clusters that correspond to a partition [*p*_1_,*p*_2_,…*p*_*l*_] with *l* unique DNA fragments is:

7 $$ P([p_{1},p_{2},\ldots p_{l}]) C_{l0}(l) \prod_{t=1}^{l} {\lambda}_{p_{t}}   $$

where *P*([*p*_1_,*p*_2_,…*p*_*l*_]) is the number of distinct permutations of the elements in the partition. For example, the expected frequency of clusters corresponding to the partition [2,1,1] is 3×*λ*_2_ *C*_30_(3). The expected frequency of *C*_*i*0_(*j*) for 1<*j*<*i* can be estimated by enumerating the integer partitions of *i* with *j* elements and summing their expected frequencies using Eq. (7). Therefore, we have have two unknown variables (*C*_*i*0_(1) and *C*_*i*0_(*i*)) and two Eqs. (3 and 5) for the observed counts *C*_*i*0_ and *C*_*i*_. We can solve these two linear equations to estimate the two variables and estimate *λ*_*i*_. The estimates of *λ*_*i*_ and *C*_*i*0_(*i*) can then be used to solve the equations for clusters of size *i*+1. The full procedure for estimating the PCR duplication rate is as follows:

1. calculate the cluster counts (*C*_1_,*C*_2_,*C*_3_,…*C*_*n*_) from the sorted and aligned reads (*n* is the largest cluster size)
2. calculate the cluster counts *C*_*ik*_ (1≤*i*≤*n* and 0≤*k*≤*i*/2) using reads that overlap heterozygous variants
3. calculate *U*_2_ using Eq. (1) and ${\lambda }_{2} = \frac {2\times C_{21}}{C_{1}} $
4. for i = 3 to n
  calculate estimates for *C*_*i*0_(*j*) for 1<*j*<*i* using the integer partitions for *i* and Eq. (7)
  solve Eqs. (3) and (5) to estimate *C*_*i*0_(1) and *C*_*i*0_(*i*)
  calculate $U_{i} = \frac {\sum _{j} j \times 2^{j-1} C_{i0}(j)} { C_{i}} $ and ${\lambda }_{i} = \frac {C_{i0}(1)}{C_{1}} $
5. estimate the PCR duplication rate using Eq. (2)

In the above procedure, clusters for which the cluster count *C*_*i*_ is smaller than a threshold (default value of 20) are ignored since it is not feasible to estimate *U*_*i*_ with small counts. This may lead to a slight underestimation of the PCR duplication rate, however, results on real data suggest that this has a minor effect. Also, solving the Eqs. (3) and (5) can result in negative values of *C*_*i*0_(*i*) or *C*_*i*0_(1). In such cases, we approximate the values as 0.

### Sequence and genotype data

For estimating PCR duplication rates on DNA and RNA-seq data, we utilized 40 samples from the GBR and FIN populations in the 1000 Genomes project [15] for analysis for which both whole-exome and RNA-seq data was available (see Additional File 1: Table S1 for the list of samples analyzed and summary statistics). Exome sequencing for these samples was done as part of the final phase of the 1000 Genomes Project at a mean depth of 65.7x using the Illumina HiSeq instrument (75–101 base pair paired-end reads) and Agilent SureSelect target enrichment [15]. The sequence reads for each exome dataset have been aligned to the reference genome (hg19) using the BWA aligner [27]. The sorted BAM files corresponding to aligned paired-end Illumina exome data were downloaded from the ftp website of the 1000 Genomes project (ftp://ftp-trace.ncbi.nih.gov/1000genomes/ftp/phase3/data/).

The Geuvadis project has performed mRNA sequencing on 462 cell lines from individuals in the 1000 Genomes Project using the Illumina HiSeq2000 platform and paired-end 75 bp reads [16]. The sequence reads for the RNA-seq data have previously been aligned to the reference genome (hg19) using the GEM mapping tool [16]. We downloaded the RNA-seq BAM files for the 40 GBR and FIN samples from the EBI express ftp site. A VCF file with Omni genotype data was also downloaded from the 1000 Genomes ftp site (ftp://ftp.1000genomes.ebi.ac.uk/vol1/ftp/release/20130502/supporting/) and individual VCF files were created using heterozygous genotypes. These VCF files were used for estimating the PCR duplication rate for the exome and RNA-seq data for each individual. To assess the PCR duplication rate on datasets prepared using the Nextera library preparation protocol, a set of 12 exome datasets (aligned BAM files and variant calls in VCF format) was downloaded from the Illumina BaseSpace repository. These datasets correspond to 4 replicates of each of the three samples NA12878, NA12891 and NA12892 (a trio from the CEU population).

### Variant calling and filtering

For DNA sequencing datasets (e.g. exome data from the 1000 Genomes project), variants were called from sorted BAM files using the GATK (v3.3) UnifiedGenotyper tool. Heterozygous variants identified from the exome data were filtered to retain a set of high-confidence heterozygous single nucleotide variants (SNVs) that were used for the estimation of the PCR duplication rate. Unlike variant detection from genome or exome sequencing, variant calling from RNA-seq is challenging due to the complexity of aligning spliced reads correctly to the genome. Therefore, we utilized heterozygous SNVs identified from exome sequencing data and Omni genotype data to estimate the PCR duplication rate for RNA-seq data for each individual.

To reduce the impact of false heterozygous variants and variants with a strong allele bias on the estimation of the PCR duplication rate, we filtered the set of input heterozygous variants (exome or genotype based) using the read counts from the sequence data being analyzed. Variants with high coverage (read depth ≥30) for which the reference allele frequency was <0.1 or >0.9 were removed. Reads with a low mapping quality (threshold of 30 for BWA aligned reads) were not utilized for estimating the PCR duplication rate. Such reads are randomly aligned to one of the possible locations by alignment tools mkaing it difficult to reliably identify clusters of read duplicates or perform variant calling.

### Simulation procedure

Read duplicates were removed from the sorted BAM file using the picard MarkDuplicates tool to create a BAM file with unique reads only. Next, reads that overlapped heterozygous SNV sites were identified. To simulate natural read duplicates, a subset of the reads with size proportional to (1 - read duplication rate) was randomly selected and duplicate reads were randomly sampled with replacement from the selected set of unique reads [12]. Each duplicate read was assigned the reference or variant allele at heterozygous sites with equal probability. Subsequently, we simulated PCR duplicates at varying rates (0–0.4) using the same approach. The only difference was that each duplicate read was assigned the same allele at heterozygous variant sites as the original read. For each value of the PCR duplication rate, 50 replicates were simulated and the PCR duplication rate estimated using our method. We simulated data using five values for the read duplication rate due to sampling: 0, 0.1, 0.2, 0.3 and 0.4.

## Additional file

Additional file 1 Supplementary data. This is a PDF document that contains **Table S1** and **Figures S2** and **S3**. (PDF 374 kb)
