# Supplementary material for: A computational method for estimating the PCR duplication rate in DNA and RNA-seq experiments
Source: BMC Bioinformatics. 2017 Mar 14;18(Suppl 3):43. doi: 10.1186/s12859-017-1471-9 (PMC5374682; doi:10.1186/s12859-017-1471-9)
Supplement: Additional file 1 — Supplementary data. This is a PDF document that contains Table S1 and Figures S2 and S3. (PDF 374 kb) [file 12859_2017_1471_MOESM1_ESM.pdf]

Supplementary Data for "A computational method for estimating the PCR duplication rate in high-throughput DNA sequencing experiments"

Table S1: List of 40 samples from 1000 Genomes Project used for PCR duplication rate analysis (statistics from exome data)

Sample	Population	# of reads used	Read duplication rate (PE)	PCR duplication rate (PE)	Read duplication rate (SE)
HG00101	GBR	52039587	0.0779	0.074835	0.2861
HG00105	GBR	25077100	0.0462	0.043821	0.2243
HG00108	GBR	59569448	0.0706	0.067423	0.3276
HG00109	GBR	60005235	0.0607	0.057193	0.3159
HG00110	GBR	58238727	0.058	0.05482	0.3145
HG00111	GBR	44170975	0.0339	0.030792	0.2936
HG00121	GBR	47499507	0.0311	0.02873	0.2577
HG00125	GBR	43660871	0.0399	0.037193	0.276
HG00126	GBR	28716880	0.0524	0.050049	0.2408
HG00149	GBR	25935713	0.0543	0.050944	0.2365
HG00155	GBR	65528854	0.183	0.178348	0.4296
HG00158	GBR	82379994	0.1179	0.115429	0.2344
HG00179	FIN	36590418	0.1531	0.150348	0.3331
HG00180	FIN	24493629	0.0542	0.051157	0.2283
HG00182	FIN	54552252	0.0865	0.083623	0.2793
HG00185	FIN	48889024	0.0637	0.060982	0.2618
HG00187	FIN	47981811	0.0954	0.092898	0.2668
HG00188	FIN	49518818	0.0598	0.05644	0.2607
HG00189	FIN	48354999	0.0837	0.081279	0.245
HG00235	GBR	21942866	0.0516	0.048938	0.2188
HG00250	GBR	42042019	0.0335	0.031016	0.2458
HG00252	GBR	56342582	0.0439	0.04052	0.3189
HG00278	FIN	46343370	0.0344	0.031	0.3052
HG00280	FIN	54269819	0.0331	0.030494	0.2931
HG00281	FIN	45460431	0.0329	0.030283	0.2771
HG00284	FIN	47121877	0.0376	0.034822	0.2858
HG00306	FIN	22611363	0.0804	0.077717	0.2722
HG00320	FIN	47497988	0.0391	0.036132	0.2674
HG00323	FIN	46018786	0.0367	0.033742	0.2607
HG00324	FIN	42813717	0.0293	0.026942	0.2699
HG00326	FIN	52087485	0.0369	0.033908	0.2924
HG00330	FIN	47031842	0.0295	0.026379	0.2762
HG00336	FIN	46325052	0.0293	0.026685	0.2579
HG00342	FIN	43439553	0.0347	0.032149	0.2531
HG00343	FIN	40318522	0.0265	0.02412	0.2425
HG00345	FIN	45724053	0.0363	0.033868	0.2805
HG00353	FIN	43923597	0.0357	0.032749	0.2916
HG00371	FIN	32991155	0.0999	0.09636	0.3337
HG00383	FIN	43665814	0.0282	0.025679	0.2332
HG02215	GBR	58194492	0.088	0.083882	0.3777

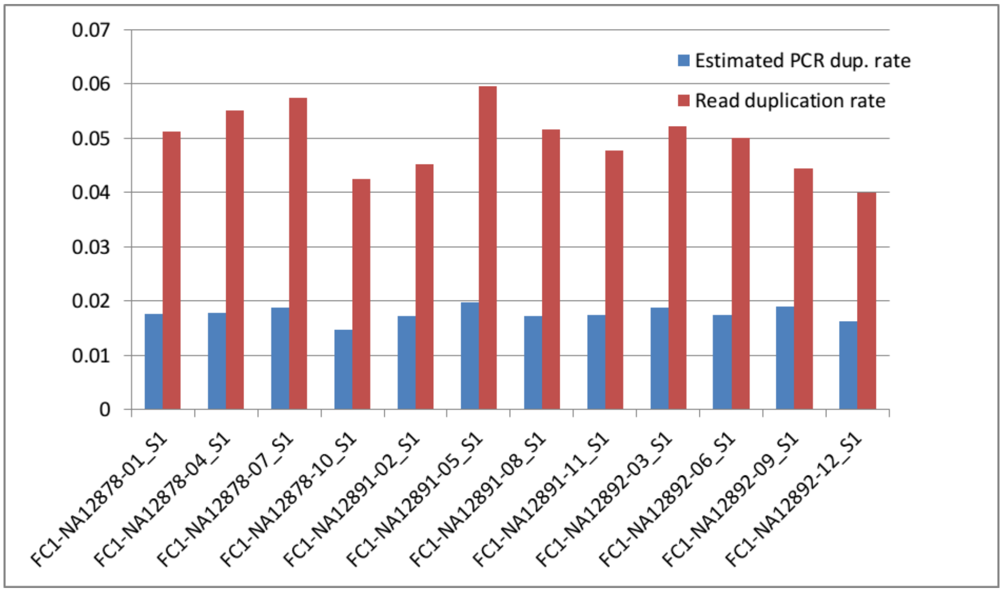

Figure S2: Read duplicate and PCR duplication rate estimates for the Illumina Nextera exome datasets

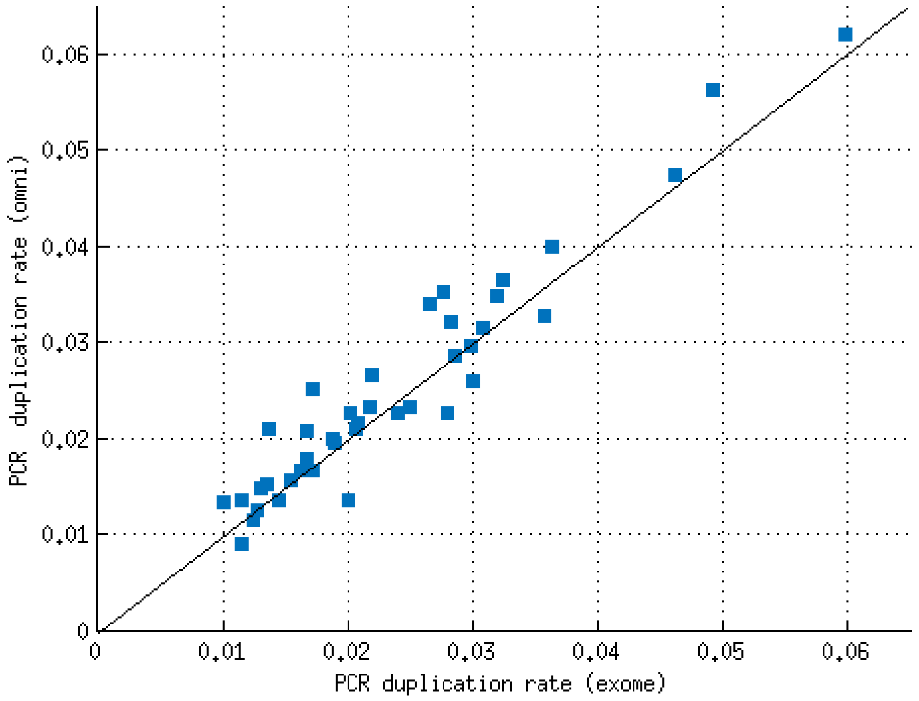

Figure S3: Concordance between the PCR duplication rate estimates for the 40 RNA-seq samples using two sets of variant calls derived from exome sequencing and Omni genotyping
